# Circulating Plasma Levels of MicroRNA-21 and MicroRNA-221 Are Potential Diagnostic Markers for Primary Intrahepatic Cholangiocarcinoma

**DOI:** 10.1371/journal.pone.0163699

**Published:** 2016-09-29

**Authors:** Camilo Correa-Gallego, Danilo Maddalo, Alexandre Doussot, Nancy Kemeny, T. Peter Kingham, Peter J. Allen, Michael I. D’Angelica, Ronald P. DeMatteo, Doron Betel, David Klimstra, William R. Jarnagin, Andrea Ventura

**Affiliations:** 1 Department of Surgery, Memorial Sloan-Kettering Cancer Center, New York, NY, United States of America; 2 Cancer Biology and Genetics Program, Sloan-Kettering Institute, New York, NY, United States of America; 3 Department of Medicine, Memorial Sloan-Kettering Cancer Center, New York, NY, United States of America; 4 Department of Medicine and Institute for Computational Biomedicine, Weill Cornell Medical College, New York, NY, United States of America; 5 Department of Pathology, Memorial Sloan-Kettering Cancer Center, New York, NY, United States of America; University of Connecticut Health Center, UNITED STATES

## Abstract

**Background:**

MicroRNAs (miRNAs) are potential biomarkers in various malignancies. We aim to characterize miRNA expression in intrahepatic cholangiocarcinoma (ICC) and identify circulating plasma miRNAs with potential diagnostic and prognostic utility.

**Methods:**

Using deep-sequencing techniques, miRNA expression between tumor samples and non-neoplastic liver parenchyma were compared. Overexpressed miRNAs were measured in plasma from an independent cohort of patients with cholangiocarcinoma using RT-qPCR and compared with that healthy volunteers. The discriminatory ability of the evaluated plasma miRNAs between patients and controls was evaluated with receiving operating characteristic (ROC) curves.

**Results:**

Small RNAs from 12 ICC and 11 tumor-free liver samples were evaluated. Unsupervised hierarchical clustering using the miRNA expression data showed clear grouping of ICC vs. non-neoplastic liver parenchyma. We identified 134 down-regulated and 128 upregulated miRNAs. Based on overexpression and high fold-change, miR21, miR200b, miR221, and miR34c were measured in plasma from an independent cohort of patients with ICC (n = 25) and healthy controls (n = 7). Significant overexpression of miR-21 and miR-221 was found in plasma from ICC patients. Furthermore, circulating miR-21 demonstrated a high discriminatory ability between patients with ICC and healthy controls (AUC: 0.94).

**Conclusion:**

Among the differentially expressed miRNAs in ICC, miR-21 and miR-221 are overexpressed and detectable in the circulation. Plasma expression levels of these miRNAs, particularly miR-21, accurately differentiates patients with ICC from healthy controls and could potentially serve as adjuncts in diagnosis. Prospective validation and comparison with other hepatobiliary malignancies is required to establish their potential role as diagnostic and prognostic biomarkers.

## Introduction

Intrahepatic cholangiocarcinoma (ICC) is the second most common primary hepatic malignancy after hepatocellular carcinoma but its age-adjusted incidence is constantly rising [1,2]. The only potentially curative treatment is complete resection, which offers a median overall survival approximating 30 months [3–6]. Due to a nonspecific presentation, most patients are diagnosed at an advanced stage precluding resection. Moreover, clinical and radiologic differentiation from other primary liver tumors (both malignant and benign) and from metastatic disease can be challenging and the value of percutaneous diagnostic biopsy is uncertain [7]. Identifying non-invasive and reliable biomarkers that aid in early diagnosis would represent a significant advance, especially in high-risk populations. The ideal diagnostic biomarker is readily measurable, minimally invasive, reproducible, and highly accurate at identifying the disease at hand. There are no reliable diagnostic biomarkers for ICC currently endorsed in clinical practice, and the only established prognostic factors are pathologic features requiring invasive tumor tissue procurement.

MicroRNAs (miRNAs) are small non-coding RNAs that modulate the gene expression at the post-transcriptional level in a sequence-specific manner. Functional studies have shown miRNAs to participate in almost every cellular process including apoptosis, proliferation and differentiation by directly modulating the expression of tumor suppressor genes and oncogenes [8–10]. A potential diagnostic, prognostic and therapeutic value of miRNA expression profiles in ICC has been reported [11–13]. Most recently, Zhang et al reported a microarray study where they identified a 30-miRNA signature which distinguished ICC from normal biliary epithelium, and a 3-miRNA signature that accurately predicted prognosis in patients diagnosed with ICC with an area under the curve (AUC) of 0.747. [14]

Certain miRNAs are stable and easily measurable in serum and plasma and therefore hold the potential to be ideal cancer biomarkers [15–17]. Promising results have been seen using circulating miRNAs as predictors of outcome in various malignancies [18–22]. However, there is a paucity of studies evaluating circulating miRNAs specifically as markers of ICC.

The goal of the current study was to find circulating markers of ICC. To this end, we used historical FFPE (formalin fixed paraffin embedded) tumor samples and deep sequencing techniques to determine the miRNA expression profile of ICC in two independent cohorts of patients (exploratory and validation). Furthermore, aiming to identify circulating miRNAs that are abnormally elevated in the setting of ICC, deregulated miRNAs were measured in plasma from an independent group of patients with ICC to establish feasibility as potential circulating diagnostic markers of the disease.

## Methods

### Patient samples

Authorization was obtained from our institutional review board (IRB) and the human biospecimen utilization committee (HBUC). The hepatopancreatobiliary surgery database was queried to identify patients with ICC, and this query was crossmatched with the Department of Pathology’s tissue procurement services (TPS) database to identify availability of tissue. Patients with available tumor tissue, tumor-free liver parenchyma, and in whom preoperative plasma had been collected were identified. All patients had provided informed consent for tissue banking. Tumor tissue and non-tumoral hepatic parenchyma were collected during exploratory laparotomy for liver resection in patients with resectable disease, or hepatic arterial infusion (HAI) pump placement for those with unresectable disease at presentation. Most patients with unresectable ICC were treated with HAI of floxuridine, with or without intravenous bevacizumab as part of two phase II clinical trials previously published [23,24]. For studies on circulating miRNA, plasma samples were obtained from a control cohort of healthy volunteers over the age of 40 with no diagnosis of cancer. Ten milliliters of whole blood from patients and controls were collected in ethylenediaminetetraacetic acid (EDTA) coated tubes and spun down within 30 minutes of collection to retrieve the plasma. Blood collection was performed within seven days before resection in resected patients or before treatment initiation in unresected patients. Plasma was stored in 1ml aliquots and preserved at -80 degrees Celsius until analysis. Solid tissue obtained from TPS were collected directly from the operating room or pathology suite and stored either as formalin fixed paraffin embedded (FFPE) blocks, or frozen at -80 degrees Celsius according to established protocols and keeping strict track of processing times [25]. To ensure adequate RNA integrity, we selected specimens with less than 2 hours from collection to freezing.

### RNA extraction

Whole-section of frozen tumor samples were pulverized in liquid nitrogen and homogenized in Trizol solution followed by RNA isolation according to the manufacturer's instructions (Life technologies). Whole-section curls were obtained from FFPE tissue blocks without microdissection and processed after deparaffinization steps using the RecoverAll kit (Life technologies). RNA was extracted from plasma samples following the mirVana platform as previously published [26,27]. Three steps of phenol/chloroform purification were added to increase purity of the RNA samples.

### Small RNA sequencing

Small RNA libraries were generated using the TruSeq kit from Illumina and subjected to deep sequencing using the Hi-Seq 2000 Illumina platform. The reads were then mapped to the human genome (hg19 build), and the normalized expression of each miRNA was determined using the DESeq software [28].

Expression levels of individual miRNAs were compared between cholangiocarcinoma and companion non-tumor bearing liver samples. miRNAs with log expression change p-value < 0.0001 by paired t-test, at a false-discovery rate (FDR) of 1%, were considered differentially expressed.

### Isolation and quantification of circulating miRNAs

For normalization purposes, plasma samples from healthy controls and patients with ICC were spiked with synthetic RNA oligos corresponding to C. elegans miR-39, miR-54, and miR-213 at a final concentration of 10 fmol/ml prior to RNA extraction. Expression levels of the selected human miRNAs were determined by RT-qPCR using specific Taqman primers (Applied Biosystems) and normalized to expression of the “spiked-in” C. elegans miRNAs, as previously described [26]. The discriminatory ability of the evaluated plasma miRNAs between cholangiocarcinoma patients and healthy volunteers was evaluated with receiving operating characteristic (ROC) and area under the curve (AUC) analysis.

### Statistical analysis

Differences of miRNA expression between groups were calculated by the t-test or the Mann-Whitney U test as appropriate. All tests were two-tailed. A value of P < 0.05 was considered to indicate a statistically significant difference. Concordance index was calculated using receiver operating characteristic (ROC) curves. All analyses were performed using Stata/IC 12.0 (StataCorp LP, College Station, TX). Graphs were created using the GraphPad Prism 6.0 software (San Diego, CA, USA).

## Results

### Comparison of small RNA libraries obtained from FFPE and frozen specimens

**Fig 1:**
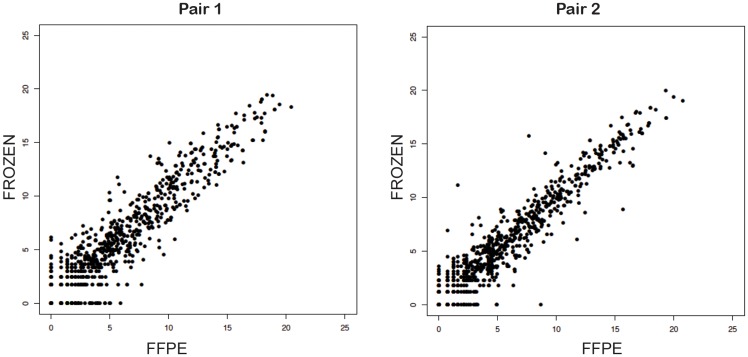
Representative scatter plots of normalized miRNA expression showing the correlation between FFPE and frozen paired tumor samples.

To determine whether small RNA sequencing from FFPE archival samples could provide reliable results, we first generated and sequenced small RNA libraries from five ICC patients in whom both frozen and FFPE samples were available thus allowing a paired comparison. The FFPE blocks RNA yield ranged between 46 and 52 ng/ml with RNA integrity numbers (RIN) between 4.3 and 7.2. Fig 1 shows two representative pairs of samples showing adequate correlation between FFPE and frozen tissue (Spearman’s pairwise correlation: 0.66, 0.91, 0.78, 0.75, 0.43; all *P* < 0.0001- t test), indicating that high-quality small RNA sequencing data can be obtained from FFPE.

### Sequencing and identification of candidate biomarkers

**Fig 2:**
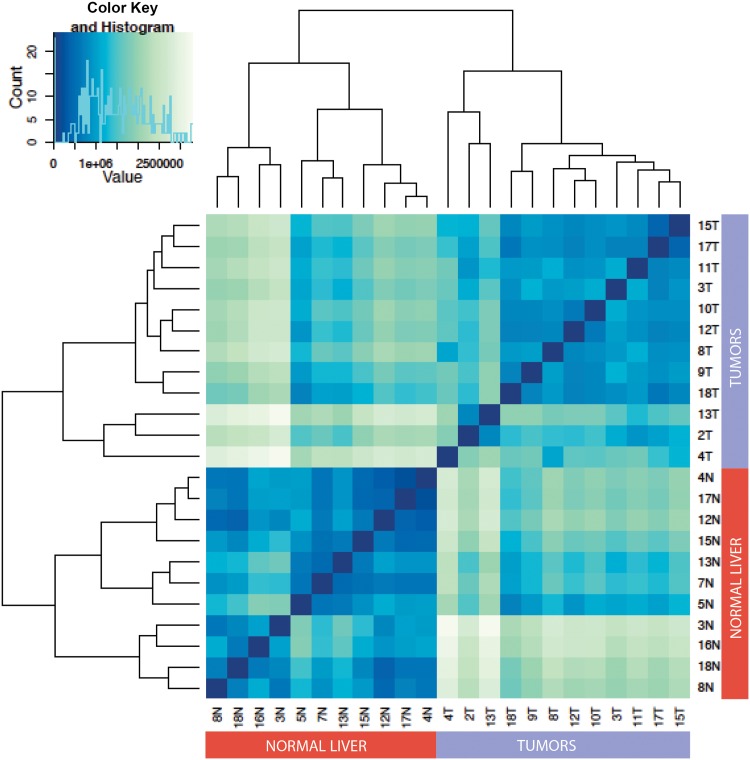
Correlation matrix heatmap showing the Euclidean distance between non-tumor bearing liver and ICC samples. Darker color indicates stronger correlation.

We extracted and sequenced small RNAs from 12 additional FFPE ICC and 11 tumor-free liver samples. On average we obtained 9.5 x 10^6^ reads mapped to known miRNAs per sample (min = 8.2 x 10^5^ reads; max = 18.7 x 10^6^ reads). Unsupervised hierarchical clustering using the miRNA expression data showed clear grouping of ICC specimens vs. normal liver (Fig 2).

**Fig 3:**
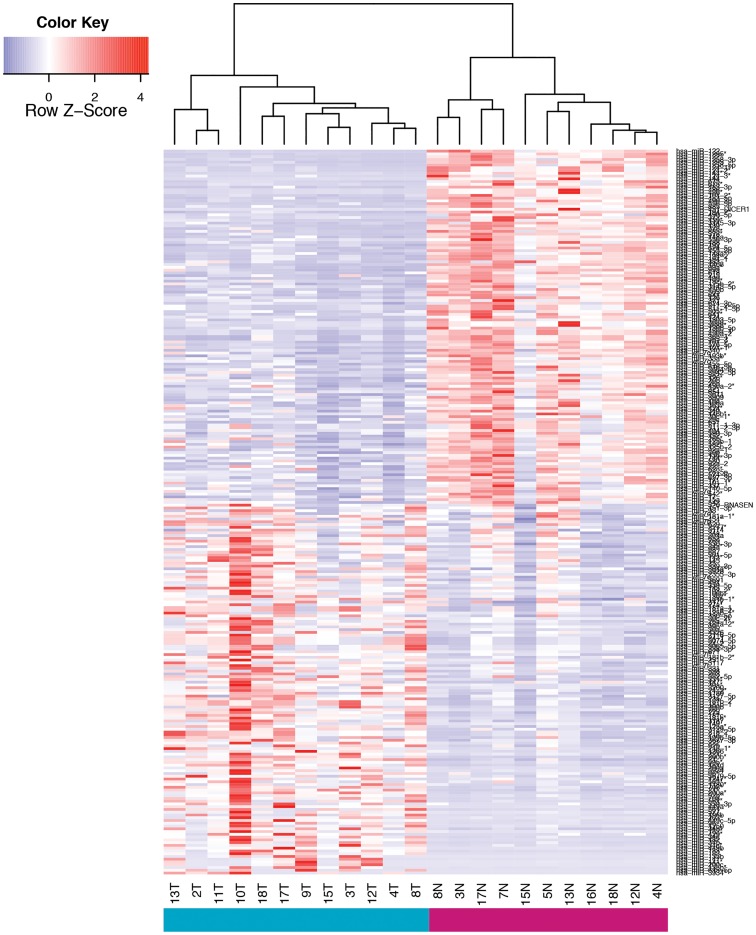
Heat map of the differentially expressed miRNAs (adjusted p value <0.001) between ICC and non-tumor bearing liver FFPE samples.

**Fig 4:**
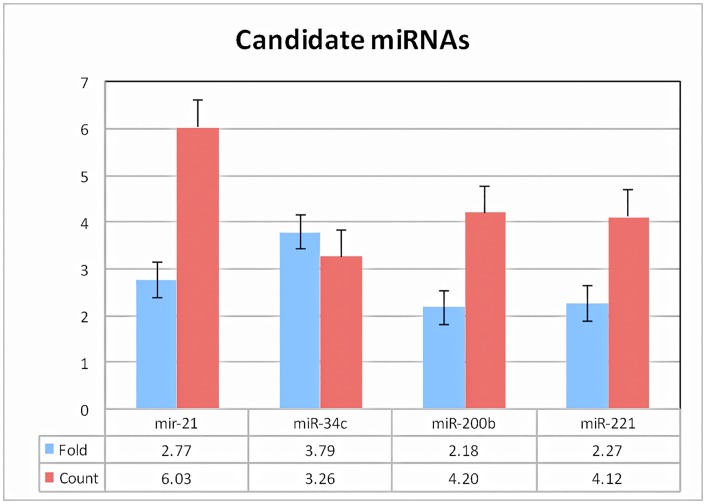
Selected miRNAs based on the highest degree of up regulation (fold change, blue columns) and absolute expression counts (red columns). Fold change was calculated between ICC and non tumor-bearing liver. Error bars represent standard error of the mean.

The analysis of these data revealed 262 microRNAs that were differentially expressed in tumor compared to tumor-free liver parenchyma (at a false discovery rate of 1%). Of these, 134 were down regulated and 128 were upregulated (Fig 3). To increase the likelihood of identifying useful plasma biomarkers, we focused on miRNAs with the highest degree of upregulation in the tumor samples (fold-change in comparison to tumor-free liver) and that were among the most abundant in terms of absolute expression levels. Based on these criteria, we selected 4 miRNAs (miR-21, miR-34c, miR-200b, and miR-221) for further analysis in plasma (Fig 4).

### Tissue validation of overexpression and exploration of circulating miRNAs

**Fig 5:**
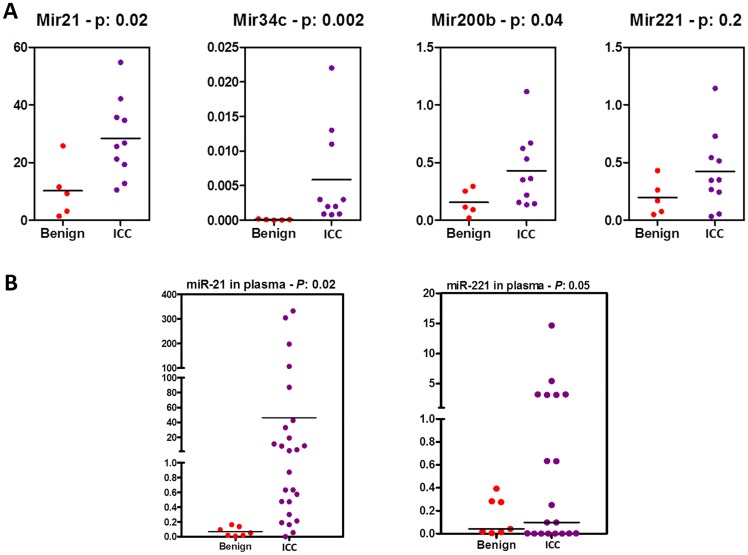
RT-qPCR validation of differentially expressed miRNAs in tissue (A) and plasma (B). (A) differential expression of miR-21, miR-34c, miR-200b and miR-221 on tissues from 10 independent patients with ICC and 10 normal liver samples. Horizontal bars represent mean value. (B) Expression levels of circulating miR-21 and miR-221 in an independent cohort of patients with ICC vs healthy controls.

An independent set of FFPE tumors (10 samples) and tumor-free liver parenchyma (5 samples) was used as a validation cohort. Real-time quantitative polymerase chain reaction (RT-qPCR) was used to confirm the differential expression of our candidate miRNAs (miR-21, miR-34c, miR-200b, and miR-221) Fig 5A. MiR-21, miR-34c, and miR-200b were also overexpressed in this cohort. Interestingly, miR-34c absolutely discriminated tumor samples from non tumor-bearing liver. While miR-221 was found in larger concentration in the ICC samples, this difference did not reach statistical significance.

**Fig 6:**
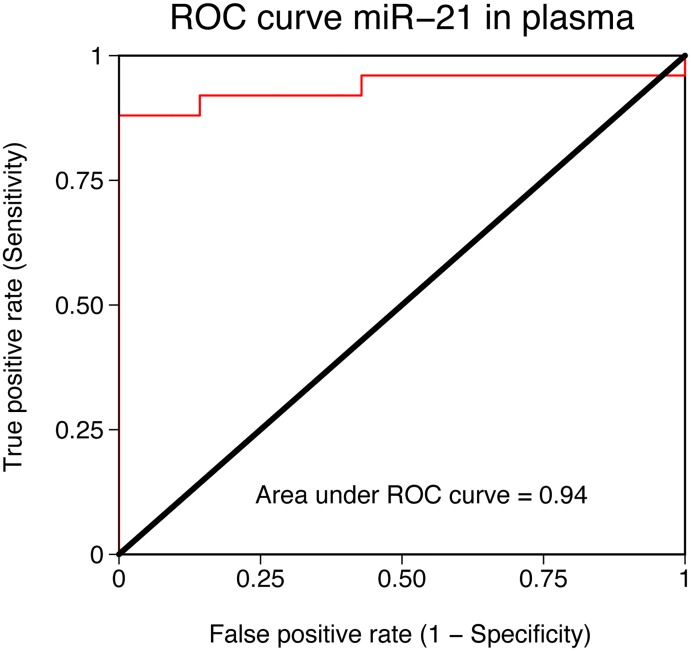
Receiver operating characteristic (ROC) curve for circulating miR-21 for the diagnosis intrahepatic cholangiocarcinoma. ROC was performed for plasma miR-21 from patients with ICC vs healthy controls. Area under the curve (AUC) for miR-21 is 0.94.

The potential role of these up-regulated candidate miRNAs as circulating biomarkers of ICC was assessed using RT-qPCR in plasma samples of 25 patients with ICC of different grades and plasma from 7 healthy controls. The normalized C_T_ (threshold cycle) values for the evaluated miRNAs in plasma from cases and controls are shown in Table 1. There was no statistical significance, regarding miR-34c and miR-200b, which found in very low quantity in the plasma of ICC patients whereas undetectable in controls. Overexpression of miR-21 and miR-221 in plasma from an independent cohort of patients with ICC is shown in Fig 5B. Discrimination between healthy controls and ICC patients, was excellent using circulating miR-21 expression (AUC = 0.94; Fig 6).

**Table 1:** Normalized expression of selected miRNAs in plasma.

microRNA	Patients		Controls		P value
	Mean	SD	Mean	SD	
***miR-34c***	0.019	0.05	Undetectable		NA
***miR-200b***	0.158	0.405	Undetectable		0.4
***miR-21***	43.881	93.421	0.070	0.062	0.0001
***miR-221***	1.657	3.584	0.147	0.164	0.05

Comparison of normalized expression of selected miRNAs in plasma of patients with ICC and healthy controls. Expression was measured with RT-qPCR and normalized with spiked-in C. elegans miR-39, miR-54, and miR-213.

### Clinicopathological correlation and prognostic value of circulating miRNAs

A total of 35 patients with clinicopathological and follow up data and were analyzed for prognostic evaluation. Descriptive data are listed in Table 2. Of these, 24 patients underwent a liver resection; they had not reached a median overall survival after a median follow-up of 28 months (IQR 10–63). Fifteen patients (65%) experienced recurrence. Circulating miR-221 expression was significantly higher in poorly differentiated tumors (n = 5) than in moderately differentiated tumors (n = 19; p = 0.016). No other tumor features such as tumor size, multiple lesions, lymphovascular invasion, nodal status, resection status correlated with miR-21 and 221 expression profiles. The remaining 11 patients had irresectable disease and were treated with hepatic arterial infusion of Floxuridine. Partial tumor response was seen in 55% and their median overall survival was 17 months (IQR 14–32). There was no association between miRNA expression levels and tumor size, multiple lesions, extrahepatic disease, or response to HAI chemotherapy. Neither of these groups displayed correlation between survival outcomes and miRNA expression levels.

**Table 2:** Clinicopathological features of ICC patients.

	Resected (n = 24)	Unresected (n = 11)	Whole cohort (n = 35)
**Age, years**	64 (13)	56 (12)	62 (13)
**Female (%)**	10 (46%)	5 (46%)	16 (46%)
**Total Bilirubin, mg/l**	1.4 (3.2)	0.9 (0.6)	1.2 (2.5)
**Albumin, g/l**	4.1 (0.4)	4.1 (0.3)	4.1 (0.4)
**Median CA 19–9, U/l (IQR)**	18.5 (61)	151 (7918)	37.5 (144)
**Median CEA, U/l (IQR)**	2.3 (1.8)	10 (132)	2.2 (2.6)
**Multiple lesions (%)**	2 (9%)	5 (45%)	9 (26%)
**Largest Tumor Size, cm**	5.6 (2.7)	8.9 (4.4)	6.6 (3.5)
**Tumor Differentiation**			
Poor	5 (21%)		
Moderate	19 (79%)		
**Lymphovascular Invasion (%)**			-
Micro	11 (45.8%)		
Macro	11 (45.8%)		
**Nodal Status (%)**			
pNx	14 (58.3%)		
pN0	8 (33.3%)		
pN1	2 (8.3%)		
**Positive Margin Status (%)**	23 (95.8%)		
**Adjuvant Therapy (%)**	6 (25%)		
**Recurrence (%)**	15 (62.5%)		
**Response to HAI FUDR (%)**			
Progression		1 (9.1%)	
Stable		4 (36.4%)	
Partial response		6 (54.5%)	

## Discussion

Using high-throughput small RNA sequencing, we have defined the spectrum of miRNAs expressed in ICC and compared them to those in normal liver. We have identified a subset of differentially expressed miRNAs—namely, miR-21, miR-34c, miR-200b, and miR-221. Furthermore, we have established that miR-21 and miR-221 are highly upregulated in ICC tumor tissue and can be detected in plasma from patients with ICC at higher concentrations compared to healthy individuals, thus suggesting their potential as diagnostic markers.

MiR-21 is known as a commonly deregulated microRNA in a variety of malignancies including lung, gastric, esophageal, colorectal, breast, prostate, pancreas, renal, glioblastoma, hepatocellular carcinoma, among others [12,21,29–40]. MiR-21 expression levels in tissue have been shown to adequately segregate cholangiocarcinoma tissue samples from normal tissues [12,41,42]. Furthermore, miR-21 has been shown to be a potential diagnostic circulating biomarker in different solid malignancies. [43] However, to our knowledge there are no previous reports showing that this miRNA is of diagnostic value in the plasma from patients with ICC.

In our patient population, among the 4 miRNAs that were selected as diagnostic candidates, miR-21 showed the highest absolute expression, while still maintaining a nearly 3-fold expression change in comparison to tumor-free liver parenchyma. This difference was maintained in the plasma samples with a striking and statistically significant difference between patients and healthy controls. When plotted in a receiver operating characteristic (ROC) curve to asses its ability as a diagnostic marker, miR-21 displayed an elevated concordance index (AUC: 0.94) which underscores its potential role as a diagnostic marker for patients with intrahepatic cholangiocarcinoma. Our findings are in line with those recently reported by Wang et al [44], in their experience, serum miR-21 segregated patients with ICC from healthy controls with an AUC: 0.94.

MiR-21 is established as an oncogenic microRNA (oncomiR) [45]. Potential mechanisms include PTEN (phosphatase and tensin homolog) tumor suppressor gene downregulation [46,47], decreasing the Bax/Bcl-2 ratio and caspase-3 activity thus negatively modulating apoptosis [48], as well as translational repression of the tumor suppressor PDCD4 (programmed cell death 4) [42,49] and downregulation of TIMP3 (tissue inhibitor of metalloproteinases 3) which is thought to function as an inhibitor of metastasis [42]. In the study by Wang et al, mechanistic roles for miR-21 were identified whereby its inhibition resulted in suppression of ICC cell proliferation in vitro and in vivo by induction of cell cycle arrest and apoptosis. They also identified PTEN, in addition to PTPN14 as functional targets of miR-21. [44] Moreover, miR-21 overexpression has been associated with increased invasiveness and ability to metastasize in cholangiocarcinoma cell lines [50]. While the latter suggests a possible prognostic role for miR-21 in ICC, there is currently no prospective data on large clinical samples to support this hypothesis. In the current study we identified no correlation between oncologic outcomes and the expression levels of these miRNAs, however, our samples size, and the heterogeneity of treatments over time limit the power of this observation.

Similarly, circulating miR-221 was differentially expressed between ICC and healthy patients. It has been reported that miR-221 is associated with a variety of malignancies including bladder, gastric, hepatocellular carcinoma, non-small cell lung cancer, but has not been reported as extensively as miR-21overexpression in ICC [18,51–53]. Potential roles of miR-221 have been reported in many cancers such as hepatocellular carcinoma formation in cirrhotic liver by targeting the tumor suppressor DNA-damage inducible transcript 4 (DDIT4), modulating the mTOR pathway, or in bladder cancer cells by modulating p53 upregulated modulator of apoptosis [54,55]. Interestingly, while miR-221 is generally considered an oncogenic miRNA, it has been recently reported as having tumor-suppressive effects in certain non small cell lung cancer cell lines by potentially inducing intra-S-phase arrest and/or apoptosis [56].

Given the oncogenic or tumor-suppressor activity of various microRNAs in different malignancies [9,57,58], several experimental approaches to profile miRNA expression in tissue samples and in biological fluids have been reported [26,27,59]. The most popular, due to the relative low cost and the limited technical complexity, are microarray based approaches and quantitative-RT-PCR methods. These methods however have important limitations. First, the analysis is limited to small RNAs for which specific probes are available, thus preventing the discovery of novel miRNAs. In addition, the cross hybridization between closely related miRNAs, often limits the specificity of the assays. Finally, these techniques do not allow the identification of base-substitution or deletion/insertion in the small RNAs, thus severely limiting the opportunity to identify unknown mutations with oncogenic potential.

In the current study, we avoided these limitations by performing an unbiased assessment of the microRNA profile of paired clinical samples of ICC and normal liver tissue using direct next-generation sequencing. Because the relative abundance of a miRNA is directly proportional to the number of sequence reads mapping, and because miRNAs differing at a single base position can be easily distinguished using this approach, the results of these experiments provide a highly detailed map of the miRNAs present in the samples analyzed.

One of the advantages of miRNAs as potential biomarkers is their high stability in body fluids, enabling their use as non invasive diagnostic and prognostic tools. Increased circulating levels of some of the microRNAs that we identified in our study have been found to be differentially expressed in patients affected by other tumor types, including breast (miR-21), esophageal (miR-21), gastric (miR-21) and lung cancer (miR-221), melanoma (miR-221), hepatocellular carcinoma (miR-21 and miR-221) [12,18,20–22,60]. Hence, extrapolating these miRNAs as exclusive diagnostic biomarkers of ICC in the general population might prove to be inaccurate, especially to differentiate ICC and hepatocellular tumors. Accordingly, further investigations are warranted for validation, notably for screening populations at risk of ICC with primary sclerosing cholangitis, primary biliary sclerosis, viral hepatitis, liver cirrhosis or parasitic biliary disease.

In clinical practice, it is often challenging to establish the nature of liver masses based on preoperative imaging and biopsies alone. Our results show that miR-21 and miR-221 detection in the plasma can discriminate between healthy individuals and patients affected by ICC. Our study has several limitations: Most notably, the ability of these markers to discriminate ICC from other malignant liver disease has not been shown. Also, given the small sample size used and the heterogeneity of treatments over the years, the assessment of the potential prognostic and/or predictive role for the identified microRNAs, or their correlation with established pathologic markers of disease severity and aggressiveness is limited. This would require a larger, prospective evaluation of patients with a range of diagnoses as biological controls.

## Conclusion

In this exploratory study, we identified a set of deregulated microRNAs in ICC. Among them, miR-21, miR-34c, miR-200b, and miR-221 are overexpressed. Furthermore, miR-21 and miR-221 are detectable in the circulation and clearly overexpressed in patients with ICC compared with healthy controls. For miR-21, these plasma expression levels accurately differentiate patients with ICC from controls and could potentially serve as adjuncts in diagnosis. Further studies, will address the need for prospective validation and comparison with other hepatobiliary malignancies to confirm the findings presented here and assess their potential role as prognostic biomarkers.
